# Comprehensive analysis of the TGF-β signaling pathway: molecular mechanisms, disease drivers, and frontiers in clinical translation

**DOI:** 10.3389/fimmu.2026.1831213

**Published:** 2026-06-04

**Authors:** Yiting Zhang, Haixia Tang, Ze Zhou, Jiabao Liao, Xiaoyu Zhou, Xin Chu, Mengqiu Shao, Caiyan Zhang, Lijuan Jiang

**Affiliations:** 1 Yunnan University of Chinese Medicine, Kunming, Yunnan, China; 2 Kunming Medical University, Kunming, Yunnan, China; 3 The First Affiliated Hospital of Yunnan University of Chinese Medicine, Kunming, China

**Keywords:** fibrosis, immune regulation, Smad/non-Smad pathway, targeted therapy, TGF-β signaling pathway, tumor microenvironment

## Abstract

Transforming growth factor-β (TGF-β) is one of the most complex and context-dependent cytokines in the immune system. Its signaling pathway regulates differentiation, functions, and microenvironment-specific adaptations of immune cells through Smad-dependent and Smad-independent pathways. In normal physiological conditions, TGF-β maintains immune tolerance, regulates T-cell fate determination, and participates in tissue repair. In pathological conditions, aberrant TGF-β signaling drives fibrosis, tumor immune evasion, and chronic inflammatory responses. In recent years, innovative technologies such as single-cell omics and spatial transcriptomics have revealed the dynamic characteristics of TGF-β signaling in different cell lineages and microenvironments. These techniques have deepened the understanding of its molecular circuits and immune regulatory networks. Therapeutic strategies targeting TGF-β, including receptor kinase inhibitors, bispecific antibodies, and nanotechnology-based delivery systems, have shown potential in disease models such as fibrosis and tumors, but still face challenges such as toxicity, side effects, and disease stage dependence. This article reviews the multidimensional mechanism of TGF-β signaling in immune homeostasis, fibrosis, and tumor progression; assesses its prospects and limitations as a therapeutic target; and proposes future directions for clinical translation through patient stratification and disease staging, precise nanodelivery, and combination therapy, providing a theoretical basis for precise intervention in immune-related diseases.

## 1 Introduction

Transforming growth factor-β (TGF-β) is one of the most functionally complex cytokines in the vertebrate immune system and plays a crucial role in maintaining immune homeostasis and regulating inflammatory responses (1). It is a key regulator of cell proliferation, differentiation, and apoptosis and is also involved in tissue repair, embryonic development, and establishment of immune tolerance (2, 3). The function of TGF-β is context-dependent. Under physiological conditions, it maintains immune balance by inhibiting effector T cells and promoting the differentiation of regulatory T cells (Tregs) (4). TGF-β regulates functional diversity of immune cells, including differentiation of T-cell subsets, polarization of macrophages, maturation of dendritic cells, and functional activation of natural killer (NK) cells through Smad-dependent and Smad-independent TGF-β signaling pathways (5). For example, at the mucosal surface, TGF-β orchestrates the critical balance between immune tolerance and inflammatory responses by directing naive CD4^+^ T cells to differentiate into immunosuppressive Treg cells to maintain self-tolerance or into pro-inflammatory Th17 cells to fight infections (6). At the tissue level, TGF-β drives the process of fibrosis in multiple organs such as the liver and lungs by activating fibroblasts and promoting excessive deposition of the extracellular matrix (7). In the tumor microenvironment, its function is more cancer stage-specific. In the early stages, TGF-β acts as a tumor suppressor to limit abnormal cell proliferation. In the later stages, it promotes immune evasion and distant metastasis by inducing epithelial–mesenchymal transition (EMT) and inhibiting antitumor immune responses (8). This review article focuses on the immune regulatory function of the TGF-β signaling pathway, its mechanisms in diseases, and the translational significance for clinical applications. We systematically summarize the following topics: 1) the molecular composition of the TGF-β signaling pathway and its regulatory mechanisms in immune cells; 2) the “biphasic role” and microenvironment-dependent characteristics of this pathway in fibrotic diseases and tumor progression; and 3) the development of biomarkers targeting the TGF-β signaling pathway and the clinical prospects of therapeutic strategies. We highlight key research progress in recent years and construct an integrated “mechanism–pathology–translation” framework to provide a theoretical basis for in-depth exploration of the role of TGF-β in immune-related diseases and investigate the feasibility and challenges of TGF-β as a therapeutic target.

## 2 Overview of TGF-β and molecular circuits

### 2.1 Ligand maturation and latent activation

The biological activity of TGF-β is precisely regulated by its unique synthesis and activation pathways. This cytokine is initially synthesized in an inactive precursor form and is cleaved by proprotein convertases such as serine proteases to form a small latent complex (SLC) composed of the latent-associated peptide (LAP) and mature TGF-β, which are non-covalently bound (9–12). Subsequently, the SLC is covalently linked to latent TGF-β binding protein (LTBP) via disulfide bonds to assemble into a large latent complex (LLC) (13), which is anchored in the extracellular matrix (ECM) to form a local signal reservoir, storing and regulating the release of TGF-β activity (14). The key to the functional activation of TGF-β lies in its specific release from this latent state, and the main mechanisms include integrin-mediated mechanical activation, proteolytic cleavage, and cell surface receptor-mediated directed presentation (15). Specifically, integrins αvβ6/αvβ8 recognize the RGD motif in LAP and apply cytoskeleton-dependent mechanical tension, which is a crucial step in regulating TGF-β activity in the epithelial barrier immune and tumor microenvironments (16). Proteases such as MMPs and plasmin achieve enzymatic activation by degrading LAP or ECM components at sites of inflammation and tissue repair (17). Notably, molecules such as GARP (LRRC32) expressed on the surface of regulatory Treg can specifically bind to latent complexes, enabling the temporal and spatial specific presentation and activation of TGF-β, thereby precisely regulating the intensity of local immune responses. This multilevel activation regulation mechanism endows TGF-β signaling with a high degree of microenvironmental dependence and provides a theoretical basis for therapeutic strategies targeting the integrin–mechanical signaling axis.

### 2.2 Receptor system and signal initiation

The initiation of TGF-β signaling relies on a precise molecular switch composed of its transmembrane serine/threonine kinase receptor system. When the ligand binds to the type II receptor (TβRII), the constitutive kinase activity of the type II receptor rapidly phosphorylates and activates the type I receptor ALK5, forming an activated receptor complex (18). It is noteworthy that the receptor combination has significant context specificity: in immune regulation and fibrosis processes, ALK5 is the dominant type I receptor (19), while in the vascular endothelial environment, ALK1 inhibits the binding of ALK5 by competing with TβRII, leading to a completely different gene expression program (20). TβRIII acts as an auxiliary receptor and further finely regulates the initiation threshold of the signal through enhancing ligand capture efficiency and specificity (21). The intracellular transport pathway after receptor activation is a key hub for precisely regulating the strength, duration, and functional specificity of TGF-β signaling, where the endocytic pathway mediated by clathrin is mainly responsible for receptor recycling and signal continuation (18), while the endocytic pathway mediated by lipid rafts/caveolae tends to degrade the receptor, playing distinct roles and jointly constituting an early sorting mechanism that determines the fate of the signal (22). The intracellular transport pathway after receptor activation is a key hub for precisely regulating the strength, duration, and functional specificity of TGF-β signaling (8). Additionally, E3 ubiquitin ligases such as SMURF, ubiquitination, glycosylation modifications of the receptor, and membrane microdomain localization of lipid rafts, together constitute a multidimensional regulatory network for signal strength. This receptor combination and selective utilization of endocytic pathways constitute the key molecular mechanism explaining the generation of different biological effects of TGF-β signaling in different cellular contexts (23).

### 2.3 Smad axis signaling

As the core pathway of TGF-β signal transduction, Smad protein complexes precisely couple extracellular signals with nuclear gene transcription (24). The activated ALK5 receptor specifically phosphorylates the C-terminal SSXS motif of Smad2/3 (25), thereby triggering the formation of a trimeric complex between receptor-regulated Smads (R-Smads) and the common mediator Smad4. The trimeric complex is transported into the nucleus through the nuclear pore complex (26). In the nucleus, the Smad complex recognizes specific DNA sequences such as the Smad-binding element SBE in the promoter region of target genes through the MH1 domain. The specificity of its transcriptional regulation is highly dependent on the collaboration with cell-specific cofactors (27). For example, in Tregs, the interaction between Smad3 and FOXP3 transcription factor is crucial for establishing immunosuppressive functions (28). In fibroblasts, the binding of Smad3 to factors such as AP-1 drives the expression of fibrosis-related genes (29). It is particularly noteworthy that the phosphorylation modification of the linker region of Smad proteins (mediated by kinases such as ERK and CDK) acts as an important signal integration platform. Without altering the total flux of Smad, it can significantly alter the preference for target gene selection (30, 31). At the same time, inhibitory Smad7 establishes a classic negative feedback loop by competitively binding to the activated ALK5 receptor and recruiting the SMURF ubiquitination system (32), preventing excessive signal amplification or out-of-control. This multilevel regulatory mechanism ensures the precise and temporal control of Smad signals in maintaining immune homeostasis and tissue repair (33).

### 2.4 Non-Smad signal hubs

**Figure 1:**
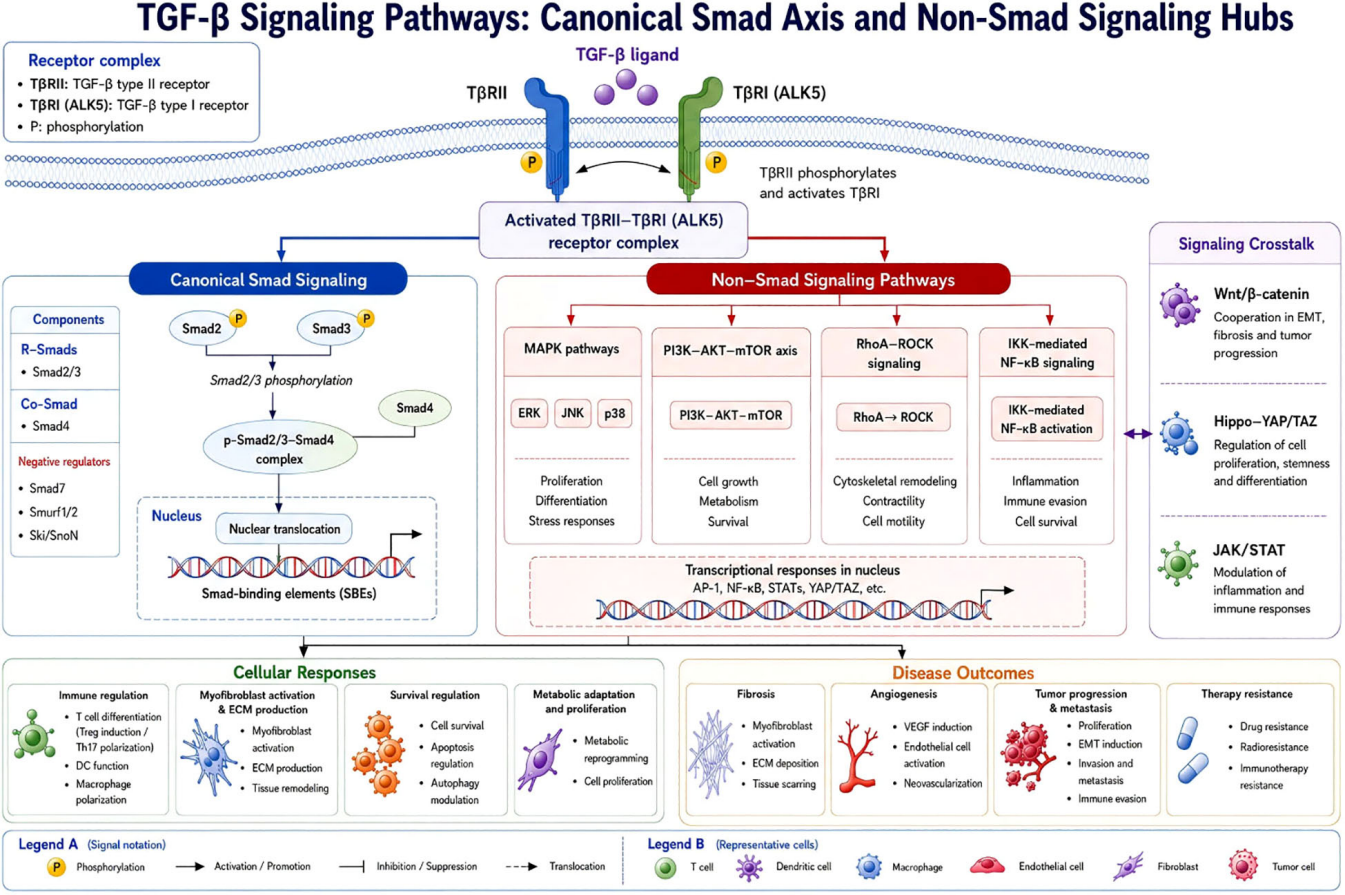
Canonical Smad and non-Smad signaling pathways downstream of TGF-β activation. TGF-β signaling is initiated by ligand binding to the TβRII–TβRI (ALK5) receptor complex, leading to activation of both canonical Smad-dependent signaling and non-Smad signaling pathways. In the canonical pathway, receptor-mediated phosphorylation of Smad2/3 promotes formation of the p-Smad2/3–Smad4 complex, which subsequently translocates into the nucleus and regulates transcription through Smad-binding elements (SBEs). In parallel, TGF-β activates multiple non-Smad signaling cascades, including MAPK, PI3K–AKT–mTOR, RhoA–ROCK, and NF-κB pathways, thereby modulating proliferation, differentiation, cytoskeletal remodeling, metabolism, inflammation, immune evasion, and cell survival. Cross-talk with other signaling networks, such as Wnt/β-catenin, Hippo–YAP/TAZ, and JAK/STAT pathways, further contributes to the context-dependent effects of TGF-β signaling. Collectively, these signaling events regulate diverse cellular responses and pathological outcomes, including immune regulation, fibrosis, angiogenesis, tumor progression, and metastasis, as well as therapy resistance.

In addition to the classic Smad pathway, TGF-β can rapidly activate multiple non-Smad signaling pathways, and these pathways play a crucial role in determining the context-dependent nature of signal output (34). During stimulation by mechanical stress, inflammatory factors, or reactive oxygen species, non-Smad pathways are often the dominant modes of signal transduction (35, 36). For example, TGF-β initiates activation of the MAPK family members such as ERK, JNK, and p38 through TAK1 to regulate cellular proliferation, differentiation, and apoptosis (37). The activated PI3K–AKT–mTOR pathway regulates cellular survival and immune tolerance by integrating growth factor and metabolic signals (38). TGF-β also regulates cytoskeletal reorganization by rapidly inducing activation of the RhoA–ROCK pathway (39). TGF-β also activates the mechanically responsive transcriptional co-activators YAP/TAZ, thereby converting physical signals of the extracellular matrix into changes in gene expression. In the context of inflammation, cross-regulation between TGF-β and classical inflammatory signaling pathways such as NF-κB and JAK-STAT further enhances its ability to integrate signals in complex microenvironments. Furthermore, these non-Smad pathways form complex synergistic or antagonistic networks with the Smad pathway, thereby collectively determining the biological effect of TGF-β signaling (40) (**Figure 1**).

### 2.5 Encoding mechanism of context dependency

The significant diversity and context-dependent nature of TGF-β signaling stems from a multilevel context-encoding mechanism that can be conceptualized through a four-dimensional regulatory model. The first dimension is defined by cell lineage specificity. For example, TGF-β induces growth inhibition in epithelial cells (18), but regulates differentiation fate in immune cells (41) and promotes matrix remodeling in fibroblasts (42). The second dimension involves differences in receptor expression profiles, which are manifested by subtle differences in the ALK1/ALK5 ratio and TβRIII levels in different cells that determine differential selection of downstream signaling pathways (43). The third dimension is reflected in the kinetic characteristics of signal transduction. Low-dose, pulsed stimulation may promote tissue repair responses, while high-intensity, continuous activation often promotes pathological processes (44). The fourth dimension involves microenvironmental factors such as ECM stiffness, hypoxia level, and local metabolites, which significantly affect the cellular responses to TGF-β (45). These four-dimensional factors are intertwined to form a complex regulatory network. The same ligand produces distinct functional outputs in different spatiotemporal contexts. Therefore, the specific pathological environment should be considered while selecting therapeutic strategies targeting TGF-β.

### 2.6 Precise regulation of immune cells

TGF-β-mediated regulation of the immune system is manifested as a multilevel and highly plastic regulatory model. The main role of TGF-β in adaptive immunity involves maintenance of T-cell homeostasis. Importantly, immunosuppression represents one of the central functional outputs of TGF-β signaling, which is essential for maintaining immune tolerance but can be pathologically exploited in tumor and chronic inflammatory microenvironments.

**Figure 2:**
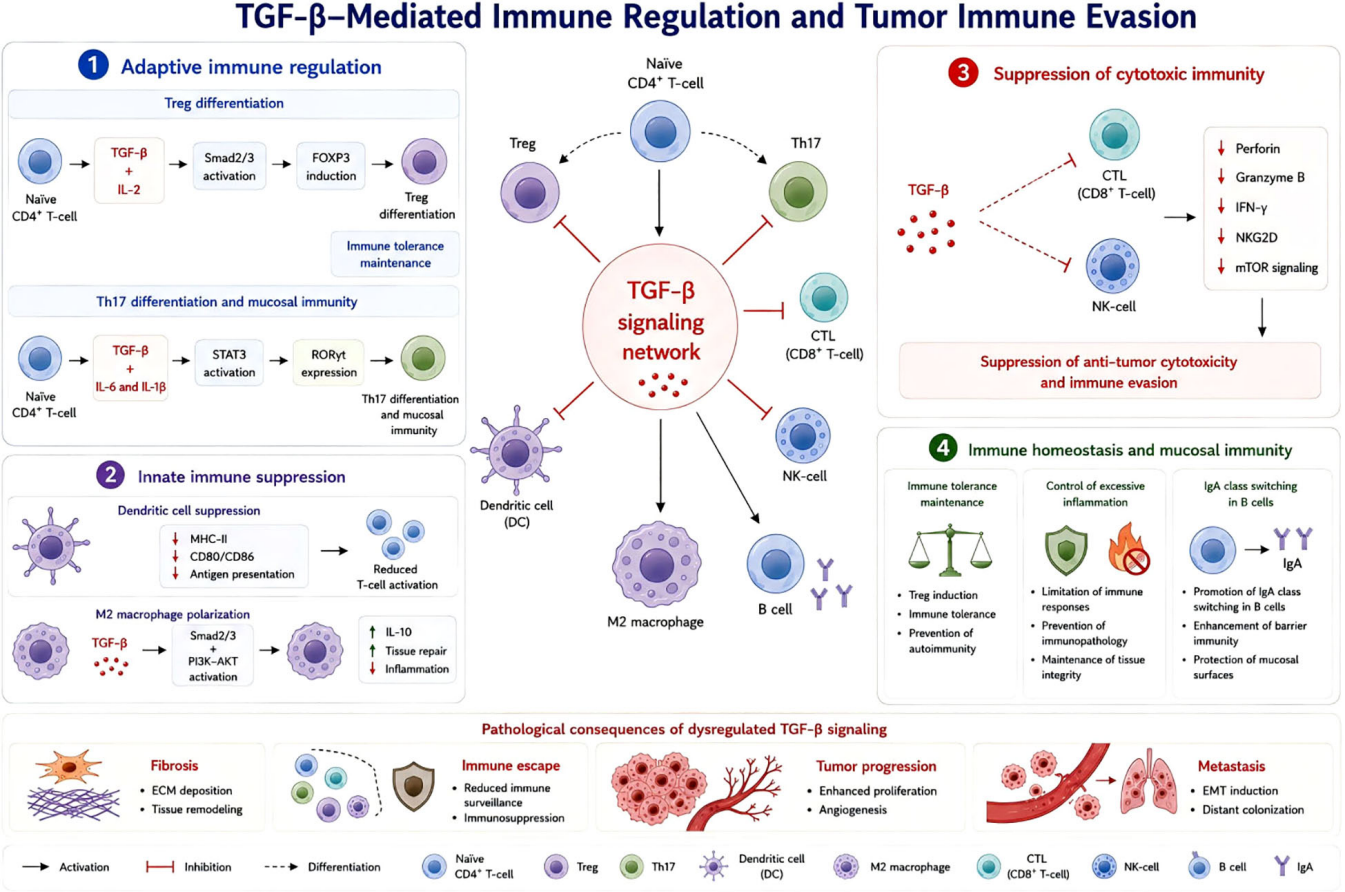
TGF-β-mediated immune regulation and tumor immune evasion. TGF-β functions as a central immunoregulatory hub that orchestrates both adaptive and innate immune responses through context-dependent signaling pathways. In adaptive immunity, TGF-β promotes the differentiation of naive CD4^+^ T cells into regulatory T cells (Tregs) through Smad2/3-mediated FOXP3 induction in the presence of IL-2, thereby contributing to immune tolerance and immunosuppression. In combination with inflammatory cytokines such as IL-6 and IL-1β, TGF-β also facilitates Th17 differentiation and mucosal immune responses via STAT3 activation and RORγt expression. In innate immunity, TGF-β suppresses dendritic cell maturation and antigen presentation by downregulating MHC-II and CD80/CD86 expression, while simultaneously promoting M2 macrophage polarization through Smad-dependent and PI3K–AKT signaling pathways, leading to anti-inflammatory and tissue repair functions. Furthermore, TGF-β contributes to tumor immune evasion by inhibiting the cytotoxic activity of CTLs and NK cells through suppression of perforin, granzyme B, IFN-γ, NKG2D, and mTOR signaling. TGF-β also participates in immune homeostasis and mucosal barrier protection by promoting IgA class switching in B cells. Dysregulated TGF-β signaling ultimately contributes to fibrosis, immune escape, tumor progression, angiogenesis, epithelial–mesenchymal transition (EMT), and metastasis.

In the presence of IL-2, TGF-β promotes differentiation of Tregs by inducing FOXP3 expression to maintain autoimmune tolerance, a process mechanistically mediated by Smad2/3 activation and direct interaction of Smad3 with the FOXP3 transcriptional complex, thereby stabilizing Treg lineage commitment and suppressive function (46). In mucosal immune defense, TGF-β drives the differentiation of naive T cells into Th17 cells when stimulated by a combination of inflammatory factors such as IL-6 and IL-1β, a process involving the cooperation between Smad signaling and STAT3 activation, highlighting the context-dependent plasticity of TGF-β-mediated T-cell fate decisions (47, 48). The fate of T-cell differentiation depends on the subtle balance of the local cytokine environment. In the innate immune system, TGF-β limits excessive immune activation by inhibiting the maturation and antigen-presenting ability of dendritic cells, partly through downregulation of MHC class II molecules and co-stimulatory signals such as CD80/CD86, thereby attenuating T-cell priming (49). At the same time, it guides polarization of the macrophage toward the anti-inflammatory M2 phenotype, a process involving coordinated activation of Smad-dependent pathways and PI3K-AKT signaling, which promotes tissue repair but may also contribute to fibrosis and tumor progression (50). However, this immunosuppressive property is exploited by cancer cells in the tumor microenvironment. TGF-β plays a key role in tumor immune evasion by inhibiting the killing function of cytotoxic T lymphocytes (CTL) and NK cells, through downregulation of effector molecules such as perforin, granzyme B, and IFN-γ, as well as suppression of mTOR signaling and reduction of activating receptors (e.g., NKG2D), thereby impairing cytotoxic responses (51). Furthermore, TGF-β plays a key physiological role in mucosal barrier immunity by promoting IgA class switching in B cells (52). Therefore, TGF-β acts as a core hub for immune balance through its pleiotropic regulatory functions. Collectively, these findings indicate that TGF-β functions as a central immunosuppressive hub that integrates multiple signaling pathways to maintain immune homeostasis, while its dysregulation contributes to immune escape and disease progression (**Figure 2**).

### 2.7 Fibrotic programs and cellular plasticity

TGF-β plays a central regulatory role in tissue repair and fibrosis by promoting the activation of myofibroblasts and conversion of cellular phenotypes. TGF-β stimulates differentiation of quiescent fibroblasts, hepatic stellate cells, and pericytes into activated myofibroblasts by upregulating α-smooth muscle actin (α-SMA) expression and the secretion and deposition of large amounts of extracellular matrix proteins, especially type I and type III collagen (53, 54). TGF-β directly activates intrinsic stromal cells and also induces EMT and endothelial–mesenchymal transition (EndoMT), thereby enabling epithelial and endothelial cells to acquire a mesenchymal phenotype and further expanding the pool of myofibroblasts (55, 56). This process is significantly regulated by mechanical signals. In a stiff tissue microenvironment, integrin–FAK and TGF-β–Smad signaling pathways act synergistically to significantly enhance the fibrotic response by activating the YAP/TAZ transcriptional co-activator (57). The interaction between mechanical and chemical signals leads to a self-amplifying trend after initiation of fibrosis, thereby providing a theoretical basis for therapeutic strategies that integrate targeting of biochemical signals (TGF-β pathway) and mechanical microenvironments (tissue stiffness) (58).

### 2.8 Endogenous feedback and braking mechanisms

To ensure precise control and timely termination of signals, the TGF-β pathway forms a multilevel endogenous negative feedback mechanism. Smad7 is a critical negative regulator that is rapidly upregulated after signal activation (59). Smad7 promotes ubiquitination and degradation of the receptor complex by competitively binding to the activated receptor and recruiting E3 ubiquitin ligases such as SMURF1 and SMURF2 (60). At the transcriptional level, nuclear transcriptional co-repressors such as Ski and SnoN directly interact with the Smad complexes to inhibit their transcriptional activity. Recent studies have found that a variety of non-coding RNAs are also involved in this fine regulatory network (61). For example, miR-21 promotes fibrosis by targeting Smad7, whereas a few long non-coding RNAs (lncRNAs) form histone modification complexes and affect the accessibility to the Smad target genes (62). Moreover, these feedback mechanisms are themselves regulated by inflammatory signals and metabolic status, thereby leading to the formation of a complex adaptive regulatory network. Furthermore, these feedback mechanisms themselves are regulated by microenvironmental factors such as inflammatory signals and metabolic states, thus forming a complex adaptive regulatory network. Understanding the operating mechanism of these endogenous braking systems—for developing selective regulatory strategies for the TGF-β pathway rather than complete inhibition—is of great significance and is expected to enable the suppression of pathological responses while preserving its physiological functions (63–65).

### 2.9 Translatable readouts and evaluation systems

A comprehensive assessment of TGF-β signaling activity requires an integrated multidimensional reading system ranging from the molecular level to the system level. At the molecular level, the detection of phosphorylated Smad2/3 (especially C-terminal phosphorylation) serves as a direct indicator reflecting the activation of the classical pathway (66); the reporter gene system based on Smad binding elements (such as CAGA repeat sequences) can quantify the transcriptional activity of the pathway (67). With the development of single-cell technologies, transcriptomics analysis can precisely parse the response characteristics of different cell populations to TGF-β. Additionally, spatial transcriptomics and multiple immunofluorescence techniques further localize the signaling activity to specific tissue microenvironment structures. At the functional level, cell phenotypic readings such as Treg/Th17 balance, macrophage polarization state, and expression of fibroblast activation markers provide important information for understanding the functional consequences of the signal. In clinical translation, the detection of potential LTBPs or activated TGF-β fragments in the blood, imaging assessment of tissue fibrosis degree, and association analysis with microenvironment characteristics (such as ECM stiffness, metabolite spectrum) together constitute a comprehensive index system for evaluating the activity of the TGF-β pathway in diseases. Establishing this multilevel assessment framework is crucial for accurately identifying patient groups that may benefit from targeted therapy.

## 3 Context-dependent immune and microenvironmental regulation

The immunoregulatory function of the TGF-β signaling pathway exhibits significant context-dependent characteristics. Its final biological effect is determined jointly by cell type, tissue microenvironment, disease stage, and systemic metabolic state. This multilevel regulatory network enables TGF-β to play distinct roles in different physiological and pathological conditions. Based on the aforementioned molecular mechanisms, this section will systematically elaborate on the complex functions of TGF-β in immune cell differentiation, tumor progression, tissue repair, and the interactions between metabolism and inflammation, revealing its multiple aspects as a key regulator of the immune microenvironment.

### 3.1 Precise regulation of immune cell lineage differentiation

TGF-β, as a core regulator of immune cell differentiation, guides the fate determination of different immune cell subsets in a microenvironment-dependent manner (68). Building upon the previous research findings on receptors and signal transduction mechanisms, it is observed that in CD4^+^ T cells, the function of TGF-β is particularly prominent: under the condition of IL-2 presence, it induces the expression of the transcription factor FOXP3, promoting the differentiation of regulatory Treg and maintaining the body’s autoimmune tolerance (69); however, when combined with inflammatory factors such as IL-6 or IL-1β, TGF-β drives the differentiation of naive T cells toward the Th17 direction, enhancing the inflammatory response and mucosal barrier defense (1). This dual regulatory mechanism demonstrates the crucial role of TGF-β in maintaining immune balance. Transitioning to the innate immune system, TGF-β also plays various regulatory roles. The innate immune system mainly includes cell types such as dendritic cells (DCs) and macrophages. Under the influence of TGF-β, dendritic cells are inhibited from maturation and their antigen-presenting ability decreases, effectively limiting excessive T-cell activation (70). Macrophages, under the influence of TGF-β, polarize from the pro-inflammatory M1 phenotype to the anti-inflammatory M2 phenotype, a transformation that is crucial in tissue repair but may also be a driving factor in the fibrotic process (71–74). It is noteworthy that the inhibitory effect of TGF-β on cytotoxic immune cells (such as NK cells and CD8^+^ T cells) is beneficial for preventing autoimmune damage, while in the tumor microenvironment, cancer cells utilize it to achieve immune escape (75). Additionally, in mucosal immunity, TGF-β promotes the IgA class transformation of B cells, strengthening the barrier immune function. TGF-β’s “double-edged sword” characteristic in immune regulation requires any targeted intervention strategy to fully consider the immune microenvironment and avoid disrupting normal immune homeostasis by comprehensive suppression.

### 3.2 Biphasic regulatory mode in tumor progression

The precise regulation of immune cells has laid the foundation for understanding the role of TGF-β in tumors. During the process of tumor occurrence and development, the TGF-β signal shows a typical stage-dependent biphasic effect. In the early stage of tumors, TGF-β exerts a powerful tumor-suppressing effect by inhibiting the cell cycle process, inducing apoptosis, and maintaining genomic stability (76). However, as the tumor progresses, cancer cells often escape the growth inhibitory effect of TGF-β through mutations or epigenetic changes, while also utilizing its ability to promote invasion and metastasis. The mechanism of this functional switch is complex and has far-reaching implications (77). In advanced tumors, the TGF-β signal shifts to be dominated by non-Smad signaling pathways, driving EMT, promoting angiogenesis, and inhibiting antitumor immune responses (78). Specifically, TGF-β promotes the proliferation of Treg cells, inhibits the functions of effector T cells and NK cells, and shapes a highly immunosuppressive tumor microenvironment. Moreover, this functional switch is regulated by multiple factors, including the expression level of TGF-β receptors, the mutation status of downstream signaling molecules (such as Smad4), and mechanical and chemical signals in the microenvironment. Cancer treatment strategies targeting TGF-β should be based on precise disease staging and molecular typing; in the early stage, it may be necessary to enhance its signal activity, while in the late stage, it should selectively inhibit its promoting metastasis and immunosuppressive functions.

### 3.3 Balance mechanism between tissue repair and fibrosis

TGF-β is also a core mediator of tissue damage repair (79). After acute injury, TGF-β promotes ECM synthesis and tissue remodeling by activating fibroblasts and perivascular cells as part of the normal repair process (80). However, when the injury persists or the negative feedback mechanism is imbalanced, the repair response becomes excessive and leads to abnormal ECM deposition and destruction of tissue architecture (81). TGF-β drives fibrosis in different organs through varying mechanisms. In the liver, hepatic stellate cells (HSCs) are activated through the Smad3 pathway, whereas non-Smad signaling pathways such as MAPK synergize with inflammatory factors to drive interstitial hyperplasia in the lungs (82). In the kidneys, the process of EMT and miRNAs jointly participate in fibrosis. Mechanical signals play a key role in the process of fibrosis. Increased ECM stiffness amplifies the fibrotic effect of TGF-β through integrin signaling (83). The goal of antifibrotic therapy is to restore the normal regulatory balance of TGF-β signaling by intervening in the key pathways and molecular mechanisms that are involved in sustained signal activation, while preserving its beneficial repair functions (84, 85).

### 3.4 Regulatory role in metabolic–inflammatory cross-talk

Beyond the traditional perspective of signal pathways, recent studies have revealed that the metabolic state and inflammatory signals form a close cross-dialogue with the TGF-β pathway through multiple mechanisms. This cross-dialogue profoundly affects the immune regulatory function of the TGF-β pathway. The hypoxic microenvironment, by stabilizing the function of HIF-1α, collaborates with the TGF-β signal to enhance Th17 differentiation and the EMT process (86); metabolic products such as lactic acid, through regulating pH and the modification state of histones, alter the response characteristics of cells to TGF-β (87, 88). This cross-dialogue is organ-specific: in the liver, bile acids interact with the TGF-β signal through nuclear receptor FXR and membrane receptor TGR5, influencing the liver immune microenvironment and fibrosis process (89). Inflammatory factors such as TNF-α and IL-1β form a positive feedback loop with the TGF-β signal by activating the NF-κB/TAK1 pathway, amplifying the inflammatory response and fibrosis process (90). This multilevel interaction provides a new perspective for understanding immune abnormalities in metabolic diseases. For the intervention of the TGF-β pathway in diseases related to metabolic abnormalities, it is necessary to consider the background influence of metabolites and inflammatory factors, and a multitarget combined strategy may achieve better therapeutic effects.

### 3.5 Remodeling of the immune microenvironment and therapeutic resistance

Based on the aforementioned multidimensional regulatory mechanisms, in chronic inflammation and tumor microenvironments, TGF-β shapes the immunosuppressive microenvironment through multiple mechanisms, promoting disease progression and treatment resistance. On one hand, TGF-β promotes excessive deposition of the ECM to form a physical barrier, restricting the infiltration of immune cells (91); on the other hand, it directly inhibits the function of effector immune cells by upregulating immune checkpoint proteins such as PD-1/PD-L1 (92).

At the same time, activated cancer-associated fibroblasts (CAFs) activated by TGF-β secrete various factors (such as vascular endothelial growth factor VEGF, chemokine CXCL12), further strengthening the immunosuppressive microenvironment, forming a vicious cycle (93, 94). This multidimensional remodeling of the immune microenvironment is one of the important mechanisms of immune therapy resistance and is also the key breakthrough direction of current combined treatment strategies. Successful combined immunotherapy requires targeting both TGF-β signaling and the microenvironment barrier simultaneously, adopting a dual strategy of relieving inhibition and microenvironment modification to effectively reverse the immunosuppressive state (95).

Based on the above discussion, the role of TGF-β in immune regulation and tissue homeostasis cannot be simply summarized as “activation” or “inhibition, ” but is a context-dependent mechanism highly dependent on cell type, microenvironment signals, disease stage, and systemic metabolic state. Understanding this complexity is crucial for developing precise targeted treatment strategies. Looking forward, research needs to further integrate multi-omics data, single-cell techniques, and spatial biology methods to resolve the network regulatory mechanism of TGF-β signaling at a higher resolution, providing a theoretical basis for individualized treatment.

## 4 Core regulatory axis in organ fibrosis

**Figure 3:**
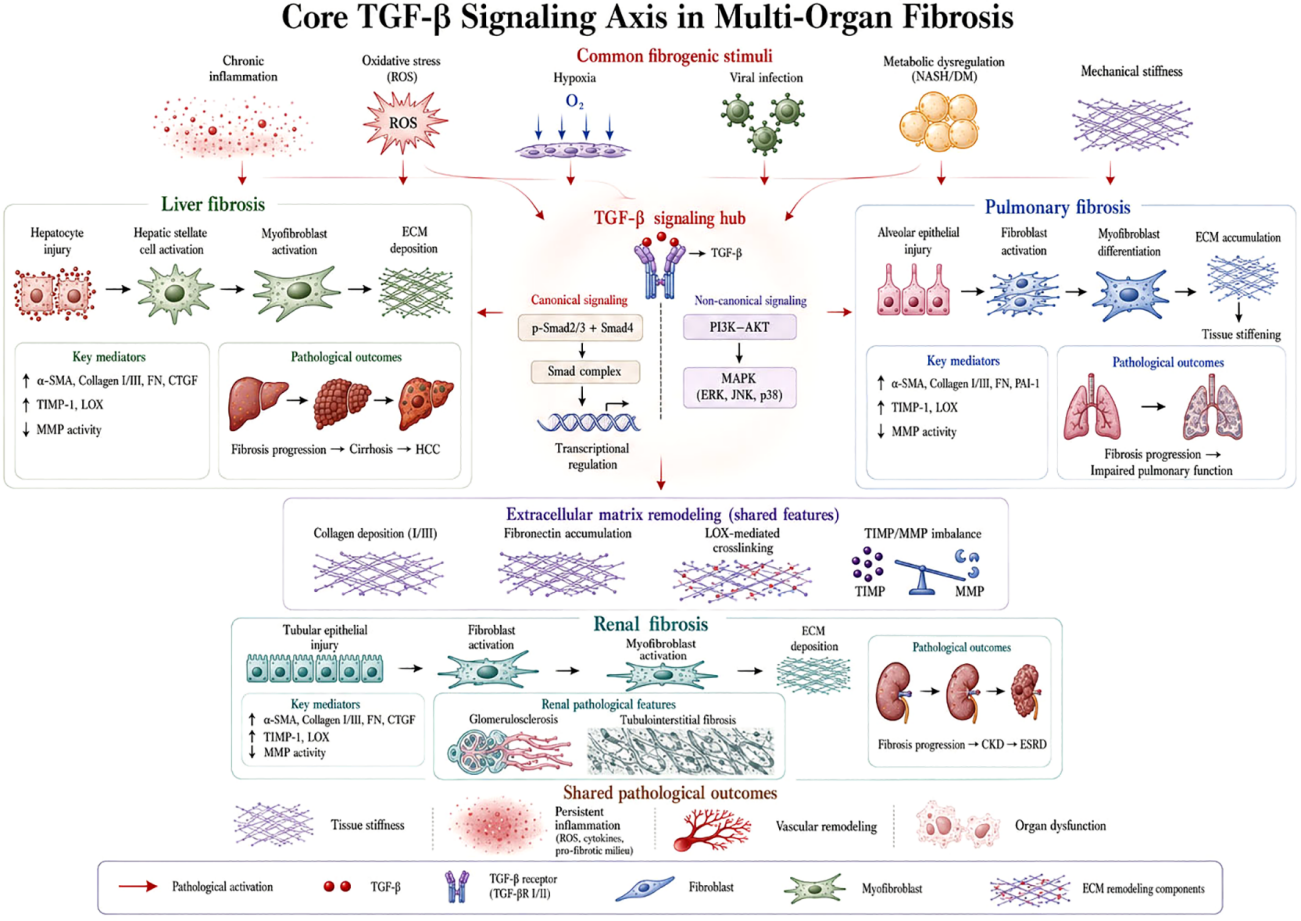
Integrated TGF-β signaling network underlying multi-organ fibrosis. Diverse fibrogenic stimuli, including chronic inflammation, oxidative stress, hypoxia, viral infection, metabolic dysfunction, and mechanical stress, converge on the TGF-β signaling hub to orchestrate fibrotic remodeling across multiple organs. Activation of canonical SMAD signaling and non-canonical pathways, including PI3K/AKT and MAPK cascades, promotes fibroblast activation, myofibroblast differentiation, and excessive extracellular matrix (ECM) deposition. In the liver, lung, and kidney, persistent TGF-β signaling drives progressive tissue fibrosis and organ dysfunction through shared mechanisms of ECM remodeling, including collagen accumulation, fibronectin deposition, LOX-mediated crosslinking, and TIMP/MMP imbalance, ultimately contributing to tissue stiffening, chronic inflammatory remodeling, vascular alterations, and irreversible functional decline.

The TGF-β signaling network is recognized as the “dominant regulator” of the fibrotic process and plays a significant role in the pathological process of fibrosis in different organs (96). Although there are significant differences in cellular composition and microenvironmental characteristics among various organs, the TGF-β-mediated fibrotic process is highly conserved and involves tissue damage, TGF-β activation, activation of fibroblast-like cells, excessive deposition of ECM, abnormal tissue structure remodeling, and loss of function (97). Guided by this consensus framework, this section systematically elaborates on the specific molecular mechanisms and regulatory networks of TGF-β in fibrosis of important organs such as the liver, lungs, and kidneys and explores the clinical translation prospects of therapeutic targeting of TGF-β signaling (**Figure 3**).

### 4.1 Multidimensional regulatory network in liver fibrosis

Liver fibrosis is the most well-studied organ fibrosis model and is predominantly driven by TGF-β-mediated signaling, in which a Smad3-dominated signaling pathway serves as the central regulatory axis (98). Specifically, binding of TGF-β to the TβRII and ALK5 complex on the cell membrane triggers C-terminal phosphorylation of Smad2/3. Subsequently, phosphorylated Smad2/3 forms a trimer with Smad4, translocates into the nucleus, and directly upregulates the expression levels of fibrosis-related genes such as α-smooth muscle actin (α-SMA) and type I collagen, thereby promoting HSC activation and extracellular matrix deposition (99, 100). This process is initiated and sustained by activated Kupffer cells and infiltrating macrophages, which secrete high levels of TGF-β and establish both autocrine and paracrine signaling loops that continuously drive HSC activation and proliferation (101). In addition, dysregulation of endogenous inhibitory mechanisms, such as reduced Smad7-mediated negative feedback, further amplifies TGF-β signaling and contributes to persistent fibrogenesis. A thorough analysis of the hepatic microenvironment reveals that multiple factors modulate TGF-β signaling outputs. The imbalance in bile acid metabolism engages in cross-talk with TGF-β through the Farnesoid X receptor (FXR) signaling pathway, influencing HSC activation (102, 103). Furthermore, reactive oxygen species (ROS) generated by oxidative stress not only facilitate activation of latent TGF-β but also amplify downstream signaling through interaction with inflammatory pathways such as NF-κB. These metabolic and inflammatory cues function primarily as modulators that enhance or fine-tune TGF-β-driven fibrotic responses (104). Notably, spatial heterogeneity within the liver also affects TGF-β responsiveness. Hepatic stellate cells in the periportal zone exhibit higher sensitivity to TGF-β stimulation than those in the pericentral region, providing a mechanistic explanation for the regional distribution of fibrosis and further highlighting the context-dependent nature of TGF-β signaling (105). Collectively, liver fibrosis can be conceptualized as a TGF-β-centered regulatory network, in which the Smad3 signaling axis acts as the primary driver, while metabolic, inflammatory, and spatial factors function as modulators that shape the intensity and outcome of fibrotic signaling. This integrated framework strengthens the mechanistic link between TGF-β signaling and liver fibrosis progression (106).

### 4.2 Mechanobiological characteristics of pulmonary fibrosis

The liver is a metabolically active organ, whereas the respiratory system focuses on gas exchange (107). In lung diseases such as idiopathic pulmonary fibrosis (IPF), TGF-β exhibits unique activation patterns and effect mechanisms. In the liver, TGF-β signaling is mediated through Smad-dependent pathways, whereas non-Smad pathways play a key role in pulmonary fibrosis. After alveolar epithelial cell injury, activated TGF-β enhances the inflammatory response by activating the MAPK signaling pathway (108). Concurrently, activated TGF-β induces cytoskeletal reorganization through the RhoA/ROCK pathway and synergizes with the mechanically sensitive transcriptional co-activators YAP/TAZ to promote EMT, leading to the massive accumulation of activated stromal cells (109, 110). The positive feedback loop between mechanical force and biochemical signaling pathways is the most significant feature of pulmonary fibrosis (111). Increased stiffness of the ECM, aggregation of integrins, and activation of focal adhesion kinase (FAK) signaling further enhance the biological activation and signal transduction of TGF-β and form a self-sustaining vicious cycle (112). Furthermore, TGF-β induces M2-type macrophages to secrete large amounts of Th2-type cytokines such as IL-4 and IL-13, which synergize with TGF-β and promote the differentiation of fibroblasts into pro-fibrotic activated fibroblasts. Together, these factors and cells form a complex and self-perpetuating fibrotic network (113, 114). Pulmonary fibrosis involves a closed-loop regulatory system, which is characterized by mechanical forces, biochemical signals, and changes in cell state, reinforcing each other. Therefore, multilevel interventions targeting the extracellular matrix-integrin-TGF-β signaling axis may be an effective treatment strategy that can overcome the current therapeutic bottleneck (115).

### 4.3 Multipathway synergistic mechanism in renal interstitial fibrosis

Interstitial fibrosis in chronic kidney disease is characterized by a complex multicomponent mechanism (116). Under continuous stimulation of TGF-β, the renal tubular epithelial cells undergo EMT and generate a large source of myofibroblasts. This process is regulated by the Smad pathway mediating transcriptional changes and non-Smad pathways such as PI3K/AKT and MAPK mediating cell survival and migration (117). Recent research breakthroughs have revealed the key role of non-coding RNA networks in renal fibrosis. MiR-21 activates TGF-β signaling by directly targeting and inhibiting the expression of Smad7 (118). LncRNA-MALAT1 induces histone methylation in the promoter region of ECM genes by binding to the EZH2 protein, thereby regulating the fibrotic process at the epigenetic level (119). Furthermore, AngII produced by the activation of the local RAS in the kidney forms a positive feedback loop with TGF-β and promotes the release of the pro-inflammatory factor IL-1β, thereby further accelerating the progression of renal tubular injury and interstitial fibrosis (120, 121). Renal fibrosis is the result of the intersection of multiple signaling axes. Interventions targeting the non-coding RNA regulatory networks and signaling pathway cross-talk may provide new directions for antifibrotic therapy.

### 4.4 Common and unique characteristics of multi-organ fibrosis

Based on a systems biology perspective, TGF-β-driven fibrosis exhibits both common rules across different organs as well as unique individual characteristics. During cardiac remodeling after injury, TGF-β promotes excessive deposition of ECM by activating the cardiac fibroblasts, leading to increased myocardial stiffness and diastolic dysfunction (122). Similar molecular mechanisms driven by TGF-β are also observed during the pathological processes of pancreatic fibrosis (e.g., chronic pancreatitis) and skin fibrosis. In-depth comparison of the basic characteristics of fibrosis in different organs reveals similarities such as Smad3 as the core signal, fibroblast to myofibroblast differentiation, and imbalance between extracellular matrix synthesis and degradation (123). However, fibrosis in different organs shows significant differences in terms of cellular origin (such as hepatic stellate cells, alveolar epithelial cells, and renal tubular epithelial cells), metabolic microenvironment (bile acids, oxygen tension, uremia-related toxins, and others), and the participation of specific immune cell populations (124, 125). These differences directly affect the pathological process and therapeutic responses. Therefore, successful antifibrotic strategies should be established under the framework of precision medicine based on the principles of combining interventions for the common targets with organ-specific adjustments. It is also necessary to formulate individualized treatment plans according to the microenvironmental characteristics of different organs.

### 4.5 Development trends and challenges of targeted therapy

**Figure 4:**
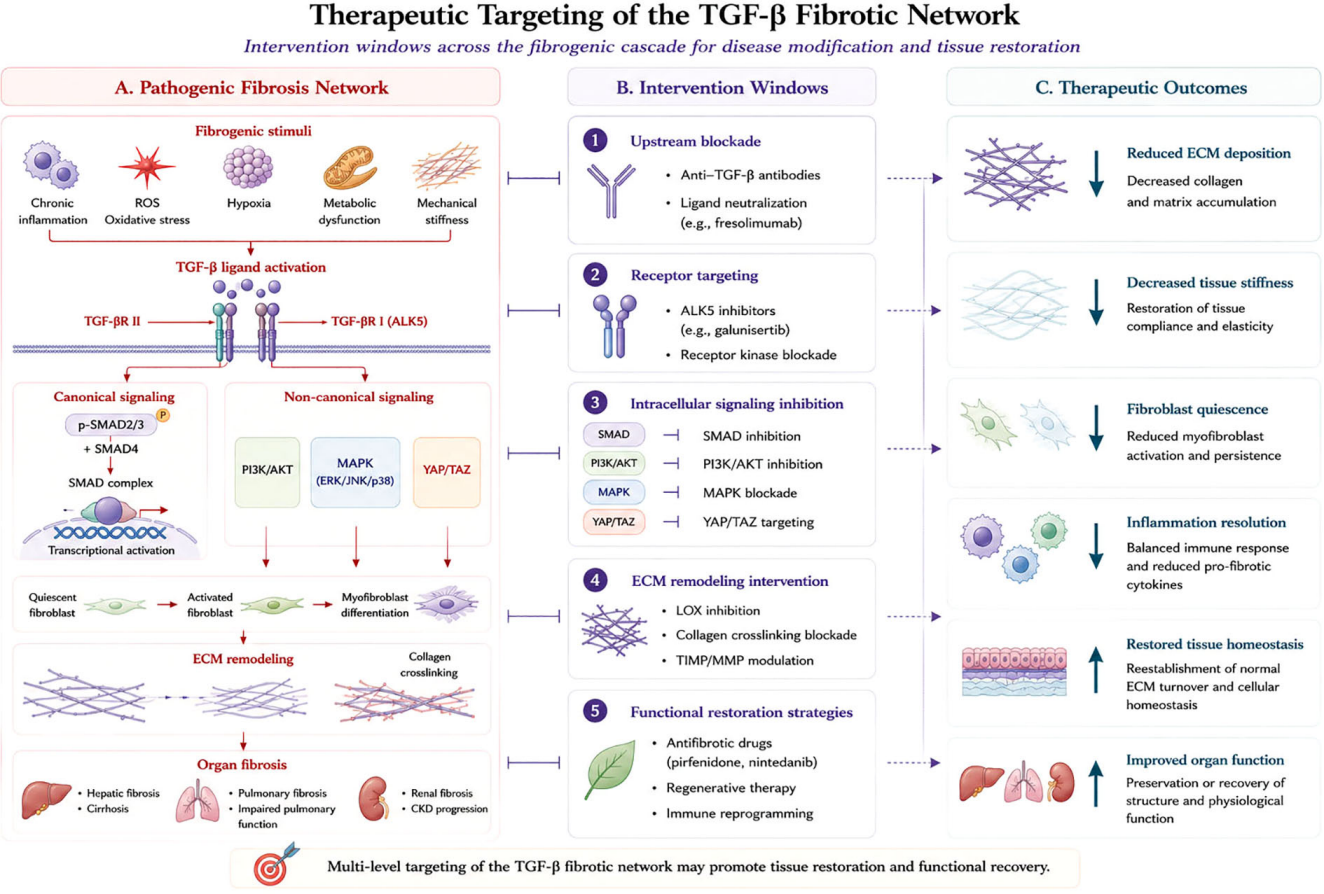
Therapeutic targeting of the TGF-β fibrotic network. Fibrogenic stimuli, including chronic inflammation, oxidative stress, hypoxia, metabolic dysfunction, and mechanical stiffness, activate the TGF-β signaling network and promote canonical SMAD-dependent and non-canonical signaling pathways involved in fibroblast activation, myofibroblast differentiation, extracellular matrix (ECM) remodeling, and organ fibrosis. Multiple therapeutic intervention windows targeting ligand activation, receptor signaling, intracellular pathways, and ECM remodeling have emerged as promising antifibrotic strategies. These approaches, together with functional restoration therapies, may reduce ECM deposition, attenuate tissue stiffness and inflammation, restore tissue homeostasis, and improve organ function across fibrotic diseases.

A variety of TGF-β pathway inhibitors are in clinical development for fibrosis, including neutralizing antibodies (fresolimumab), receptor kinase inhibitors (galunisertib), and trap molecules (soluble TβRII-Fc) (126, 127). However, the systemic application of these drugs faces significant challenges, particularly the increased risk of infection due to immunosuppression and the impairment of wound healing, which severely limit the clinical application prospects of these drugs (128, 129). To overcome this bottleneck, the current research trends mainly focus on three directions: first, developing local delivery systems based on nanoparticles and ECM-targeting peptides to achieve the specific distribution of drugs in fibrotic tissues (130); second, exploring the key time points of the “transition from acute injury repair to chronic fibrosis” for precise intervention; and third, designing combined treatment regimens of TGF-β inhibitors with anti-inflammatory, antioxidant, or metabolic regulatory drugs, enhancing efficacy through multitargeted synergy while reducing side effects (131). In summary, the success of the next-generation antifibrotic treatment will rely on systematic innovation strategies, including precise delivery, temporal control, and combined treatment, which require close collaboration and coordination between basic research, drug development, and clinical practice (**Figure 4**).

## 5 Dual roles of TGF-β in tumor progression and targeted strategies

### 5.1 Early tumor-suppressive stage

In the early stage of tumorigenesis, TGF-β exerts a strong tumor-suppressive effect through the classical Smad-dependent signaling pathway. The binding of TGF-β ligand to the TβRII-ALK5 receptor complex on the surface of the cell membrane activates the phosphorylation of Smad2/3 and formation of a transcriptional complex with Smad4, which translocates to the nucleus to regulate the expression of target genes (132). This core pathway 1) upregulates cyclin-dependent kinase inhibitors such as p15INK4b and p21CIP1, which block the activity of cyclin D–CDK4/6 complexes and cyclin E–CDK2 complexes and induce G1 phase cell cycle arrest (133); 2) promotes expression of pro-apoptotic proteins BIM and BAX and activates the mitochondrial apoptotic pathway (134); and 3) maintains integrity of the DNA damage repair mechanism to prevent genomic instability. Therefore, in normal epithelial cells and early tumor cells, the intact Smad signaling pathway constitutes an important “tumor-suppressive barrier” (135). Preclinical studies have shown that the early inactivation of the TGF-β signaling pathway (Smad4 deletion or TβRII mutation) is closely related to increased susceptibility to a variety of tumors (136). At the early stage of tumors, the intact TGF-β/Smad signaling pathway serves as a crucial mechanism for maintaining genomic stability and inhibiting malignant transformation. Its functional loss indicates the breach of the tumor suppression barrier (137).

### 5.2 Transition from a tumor-suppressor role

As the tumor progresses, the accumulation of genetic mutations and changes in the microenvironment jointly leads to a fundamental transformation in the function of the TGF-β signaling pathway. The core mechanism of this transition lies in the interaction between the impairment of the integrity of the Smad signaling pathway (such as the absence of Smad4 or mutations in ALK5) and the abnormal activation of oncogene signaling pathways (such as the RAS-MAPK or MYC pathways) (138–140). Specifically, the weakened function of the Smad pathway leads to weakened cell cycle inhibition and apoptosis induction functions. At the same time, the relative enhancement of non-Smad-dependent pathways (including MAPK, PI3K/AKT, and RhoA/ROCK) drives EMT, cell survival, and metabolic reprogramming (141, 142). Additionally, the immunomodulatory function of TGF-β undergoes a significant transformation, shifting from maintaining immune homeostasis to promoting immunosuppression (143). Studies have shown that tumor cells promote the expansion of regulatory Treg by secreting TGF-β, while inhibiting the functions of CTL and NK cells and reducing the chemotaxis of effector T cells toward the tumor site, jointly shaping the “cold tumor” immune-privileged microenvironment (144). The key events during tumor progression are not the simple increase or decrease in TGF-β expression, but the fundamental reprogramming of its signal output pattern, which is determined jointly by the genetic mutation background and microenvironment factors (145).

### 5.3 Late tumor-promoting stage

In advanced tumors, TGF-β transforms into a potent tumor-promoting factor, driving disease progression through multiple mechanisms (146). From a cellular biology perspective, TGF-β-induced EMT enhances the migration and invasion capabilities of tumor cells (147). This process involves downregulation of E-cadherin expression, upregulation of interstitial markers such as vimentin and fibronectin, and cytoskeleton reorganization (148, 149). Furthermore, TGF-β synergizes with VEGF to promote tumor angiogenesis and remodels the extracellular matrix by inducing the deposition of fibronectin and collagen (150). TGF-β also orchestrates a multilevel immunosuppressive network in advanced tumors, thereby promoting tumor immune evasion. Experimental evidence shows that TGF-β directly upregulates the expression of PD-L1 in the tumor cells and also promotes the recruitment and function of myeloid-derived suppressor cells (MDSCs); moreover, TGF-β induces the functional exhaustion of CD8^+^ T cells, thereby leading to resistance against immune checkpoint inhibitor therapy (151, 152). Furthermore, TGF-β is involved in preparing the pre-metastatic microenvironment that is favorable for tumor metastasis by regulating fibroblasts and immune cells in the distant organs to support the colonization and growth of tumor cells (153–155). TGF-β signaling in advanced tumors engineers the microenvironment to be conducive to tumor growth, metastasis, and therapeutic resistance, thereby providing a theoretical basis for combination therapy.

### 5.4 Therapeutic resistance mechanisms and clinical challenges

Activation of the TGF-β signaling pathway is an important mechanism of resistance to various tumor therapies. In terms of chemotherapy and targeted therapy, the TGF-β-induced EMT process enables tumor cells to acquire stem cell-like characteristics (156, 157). At the same time, it reduces sensitivity of tumor cells to traditional therapies by activation of survival signaling pathways such as AKT and ERK (158). In the field of immunotherapy, the TGF-β signaling pathway is one of the main factors limiting the efficacy of immune checkpoint inhibitors (ICIs). Its high activity in the tumor microenvironment is closely related to the failure of PD-1/PD-L1 inhibitor therapy (159). However, the main challenge in clinical development for systemic TGF-β inhibition includes dose-limiting toxicity and side effects such as cardiovascular toxicity and wound-healing disorders that are caused by excessive activation of the immune system (160, 161). Although TGF-β inhibitors such as galunisertib have shown promise in monotherapy, their widespread clinical application is limited by a narrow therapeutic window (162–164). Therefore, overcoming TGF-β-mediated therapeutic resistance requires the development of more selective intervention strategies rather than global inhibition of the entire pathway. This requires a deeper understanding of the spatiotemporal specificity of the TGF-β signaling pathway.

### 5.5 Innovation and development of combination therapy strategies

Based on the in-depth understanding of the dual functions of TGF-β, new combination therapy strategies are being actively explored. The most promising direction includes the combined use of TGF-β inhibitors and immune checkpoint inhibitors. Preclinical studies have shown that this combination effectively reverses the immunosuppressive microenvironment and converts “cold tumors” into “hot tumors” (165, 166). Currently, multiple clinical trials are evaluating the efficacy and safety of TGF-β/PD-L1 bispecific antibodies such as M7824 in advanced solid tumors (167). This includes the combined use of TGF-β inhibitors and anti-angiogenic drugs to improve the efficacy of drug delivery by simultaneously targeting ECM remodeling and angiogenesis and the combination with chemotherapy or radiotherapy to reverse EMT-related therapeutic resistance (168). Innovations in delivery technology also provide innovative ideas for solving toxicity issues. This includes development of tumor-targeted nanoparticle delivery systems and conditionally activated prodrugs to achieve localized and precise inhibition of TGF-β (169–171). The success of next-generation TGF-β-targeted therapy will depend on precise patient stratification, rational combination regimen design, and innovative delivery technologies, thereby achieving a shift from “systemic blocking” to “context-selective regulation.”

### 5.6 Future outlook and research directions

The spatiotemporal dynamics and functional complexity of TGF-β during tumor progression present unique challenges and opportunities for tumor treatment. The focus of future research should include the development of high-resolution signal monitoring tools, such as quantitative analysis systems for TGF-β signaling activity based on single-cell sequencing and spatial transcriptomics, to achieve real-time assessment of its functional status (172, 173). In terms of treatment strategies, it is necessary to formulate dynamic intervention plans based on tumor stage, molecular classification, and microenvironment characteristics and adopt differentiated treatment strategies at different disease stages. In the long term, a deeper understanding of the TGF-β signaling network and its role in the tumor microenvironment will drive the development of truly precise medicine. This requires us not only to focus on the TGF-β signaling status of tumor cells themselves but also to comprehensively consider the combined effects of TGF-β signaling on immune cells, stromal cells, and vascular systems, so as to design more efficient and safe treatment strategies (174, 175).

## 6 Clinical translation and precision strategies

### 6.1 Disease stage-dependent stratified therapy strategies

The context-dependent nature of TGF-β function requires an accurate matching of therapeutic strategies with the disease stage. In tumor therapy, patients in the early stage may benefit from strategies that activate TGF-β signaling to enhance the tumor-suppressive function of TGF-β because the Smad signaling pathway is intact (176). However, patients with advanced-stage tumors require strategies inhibiting TGF-β signaling to suppress its role in promoting EMT, angiogenesis, and immune evasion (177). This stage-specific function of TGF-β is also significant in fibrotic diseases. The repair function of TGF-β needs to be retained during the acute tissue repair stage, and the Smad3-driven pathological process needs to be specifically inhibited during the chronic fibrosis stage (178). The treatment strategies for autoimmune diseases are more complex, requiring a delicate balance between suppressing the pathological process and maintaining immune tolerance. Studies have shown that excessive inhibition of TGF-β may disrupt immune tolerance and lead to increased disease activity in conditions such as rheumatoid arthritis and systemic lupus erythematosus. Effective patient stratification should not be based solely on pathological diagnosis, but should take into account the disease stage, microenvironment characteristics, and pathway activity status to achieve a truly individualized treatment.

### 6.2 Precision delivery and timing control strategies

The toxicity of systemic TGF-β inhibition limits its clinical application, which makes the innovation of delivery technology a key breakthrough point. The nanoparticle delivery system can accumulate in tumor tissues by enhancing vascular permeability and retention effect (EPR effect) (179, 180). Collagen-targeting peptides can specifically deliver the inhibitor to the fibrotic area. Microenvironment-responsive precursor drugs can be activated under specific pH values, enzyme activity, or redox conditions to achieve specific release at the lesion site (181). Timing control is another important strategy. The ideal therapeutic strategy is to perform short-term blocking during the peak period of pathological signals but retaining the physiological functions of TGF-β during tissue repair stage and immune homeostasis maintenance. Bispecific platform technologies such as PD-L1/TGF-β-bispecific antibodies can target multiple pathways simultaneously, leading to enhanced therapeutic efficacy with reduced side effects (182). The combination of T cells and TGF-β blockers can overcome limitations of the immunosuppressive microenvironment on cell therapy (183). Overall, successful TGF-β-targeted therapy requires precise regulation of time, location, and dose.

### 6.3 Strategies and mechanistic synergies of combination therapy

The results of clinical trials targeting the TGF-β pathway alone suggest that combination therapy is inevitable. The most promising direction is to combine TGF-β pathway modulators or inhibitors with ICIs. Preclinical studies have shown that TGF-β inhibition can effectively reverse the immunosuppressive microenvironment, convert “cold tumors” into “hot tumors, ” and significantly enhance the efficacy of PD-1/PD-L1 inhibitors. In the field of metabolic diseases, combining TGF-β inhibitors with FXR agonists and antioxidants can target multiple players in the metabolic–inflammatory cross-talk simultaneously (184, 185). Furthermore, combining TGF-β inhibitors with anti-PDGF/VEGF drugs or ECM repair drugs can synergistically block distinct aspects of the fibrotic process (186). TGF-β-targeted therapy should be regarded as part of a systemic treatment plan and the therapeutic effect can be maximized while concurrently delaying the development of drug resistance through synergistic regulation with other pathways. Despite broad prospects, TGF-β-targeted therapy faces major challenges. Side effects such as infection risk and wound-healing disorders caused by immunosuppression limit the therapeutic window. The genetic background of patients and the heterogeneity of the microenvironment affect the therapeutic response. The lack of a standardized activity monitoring system hinders the implementation of precision medicine. Therefore, future development directions should include the 1) development of high-resolution single-cell and spatial omics technologies for *in vivo* monitoring of the TGF-β signal network, 2) establishment of humanized models and organoid platforms to simulate specific microenvironments, and 3) designing intelligent delivery systems to achieve on-demand drug release regulation.

## 7 Methodological innovation and future roadmap

### 7.1 Future perspectives on biomarker systems for TGF-β signaling

Given the highly context-dependent nature of TGF-β signaling, precise assessment of pathway activity remains a major challenge in clinical translation. Although multiple candidate biomarkers have been proposed, a standardized and clinically validated framework has not yet been fully established. Therefore, biomarker development for TGF-β signaling should currently be regarded as an emerging research direction rather than a mature clinical application system. Existing studies suggest that molecular indicators such as phosphorylated Smad2/3 nuclear translocation, Smad4 mutation status, and TGF-β receptor expression patterns may reflect pathway activity under specific pathological conditions (187). In parallel, cellular features—including Treg/Th17 balance, CAF infiltration, and immune microenvironment characteristics—may provide functional insights into TGF-β-mediated biological responses (188–190). Emerging technologies such as liquid biopsy, single-cell sequencing, spatial transcriptomics, and multi-omics integration offer promising opportunities for dynamic and non-invasive monitoring of TGF-β signaling. Circulating biomarkers, including latent TGF-β complexes, exosomal signaling molecules, and non-coding RNAs such as miR-21, have demonstrated potential value for assessing systemic pathway activity (191). In addition, imaging approaches including MRI and elastography may indirectly reflect TGF-β-driven tissue remodeling and fibrosis progression (192). However, significant limitations remain. The heterogeneity of TGF-β signaling across tissues, disease stages, and microenvironments complicates the interpretation of individual biomarkers. Furthermore, the lack of standardized detection methods and unified evaluation criteria currently limits clinical translation. Future efforts should therefore focus on establishing integrated multidimensional biomarker systems that combine molecular, cellular, imaging, and spatial information to achieve more accurate patient stratification and therapeutic guidance. Ultimately, advances in biomarker development are expected to improve the precision and clinical applicability of TGF-β-targeted interventions and facilitate the transition toward personalized medicine.

### 7.2 Integrated application of multi-omics and spatial resolution technologies

In recent years, breakthrough developments in technologies such as single-cell sequencing, spatial transcriptomics, and mass spectrometry imaging have provided powerful tools for analyzing cell-specific responses and the regional heterogeneity of TGF-β signals in complex tissue microenvironments. Specifically, single-cell RNA sequencing (scRNA-seq) can be used to identify dynamic transcriptomic changes in different subsets of immune cells (Tregs and Th17 cells) as well as stromal cells under TGF-β stimulation and also track their lineage differentiation and the evolutionary trajectory of cell states (193). Spatial transcriptomics (10× Visium and Nanostring GeoMX) further combines molecular expression profiles with *in situ* spatial information of tissues to clarify the spatial correlation between TGF-β-active regions and extracellular matrix stiffness, vascular distribution, or immune cell infiltration (194). Integrated high-throughput proteomics and metabolomics can be used to determine the activity and interaction mechanisms of signaling pathways based on the levels of post-translational modifications and metabolites and to construct a cross-molecular level regulatory network map (195, 196). The integration of multi-omics data helps to establish a “TGF-β signal activity assessment system, ” which can be used as a tool for patient stratification, efficacy evaluation, and drug resistance mechanism analysis. It can also be used to significantly improve the feasibility of therapeutic strategy and its implementation in precision medicine.

### 7.3 Testable mechanistic hypotheses and functional verification pathways

Technological advances have helped immunological research to progress from descriptive observations to mechanistic verification. This shift allows for the development of precise, testable causal hypotheses, and requires systematic verification of pathways. For example, if the hypothesis states that “the ALK5/ALK1 receptor balance and the ECM mechanical microenvironment jointly guide the TGF-β signal output, ” it can be verified by constructing conditional gene knockout models of Smad3, YAP, or TAZ using CRISPR-Cas9 gene editing to verify the roles of these individual components in the development of fibrosis or immune evasion (197). Furthermore, inducible gene knockout systems allow precise spatiotemporal control over specified gene manipulation to determine the roles of specific cell types in pathological processes (198). Furthermore, live-cell imaging in combination with biophysical technologies such as FRET biosensors and atomic force microscopy can be used to monitor dynamic changes in signals and cell behavior in real time and establish causal evidence that links molecular mechanisms with phenotypic manifestations (199). By focusing on the falsifiability of mechanisms and verifying causality, researchers can go beyond the limitations of simple correlation and provide a solid theoretical basis for target identification and intervention strategies to improve the success rate of translational research.

### 7.4 Construction of translational model systems and standardization progress

The translational potential of traditional mouse models in TGF-β signal research is limited because of significant species-specific differences in receptor distribution, immune cell composition, and other critical factors (200). Therefore, the new generation of model technologies is committed to improving its ability to predict clinical responses. Patient-derived organoids and patient-derived organ-on-a-chip technologies can retain the TGF-β response characteristics of individual tumors or diseased tissues and are suitable for drug screening and biomarker development (201). Furthermore, advanced humanized mouse models, including NSG-SGM3 and BRGSF-HIS systems, have significantly improved the translational relevance of TGF-β research by more accurately recapitulating human immune responses. These models are generated through transplantation of human hematopoietic stem cells into highly immunodeficient mice, enabling the reconstruction of functional human immune cell populations, including T cells, B cells, NK cells, and myeloid cells. In particular, NSG-SGM3 mice express human cytokines such as GM-CSF, IL-3, and SCF, which enhance human myeloid cell development and immune system reconstitution. Similarly, BRGSF-HIS models support long-term engraftment and maturation of human immune components, thereby providing a more physiologically relevant platform for studying TGF-β-mediated immune regulation, tumor immune evasion, and immunotherapeutic responses. Compared with conventional xenograft models in nude mice, which lack a fully functional adaptive immune system, humanized mouse models allow investigation of complex interactions between tumor cells, stromal components, and human immune cells within the TGF-β-regulated microenvironment. These advances substantially improve the predictive value of preclinical studies and provide important experimental platforms for precision immunotherapy research (202). Moreover, 3D bioprinting and mechanical loading systems are used to analyze the signal interaction mechanism between the mechanical microenvironment and TGF-β signals by precisely controlling ECM stiffness, fluid shear force, and cell arrangement (203). Translational models with high clinical relevance can significantly reduce the failure rate of drug development. These models provide an experimental platform with high predictive value for precision medicine and promote the rapid development of individualized treatment strategies.

### 7.5 Innovative clinical trial design and endpoint selection strategies

Clinical trials targeting the TGF-β pathway face significant bottlenecks because of patient heterogeneity and insufficient efficacy monitoring. In terms of trial design, innovative strategies should include 1) enrichment design based on biomarkers such as grouping by Smad4 deletion and TGF-β signature score to increase the proportion of responsive populations (204), 2) adaptive design that allows adjustments to the inclusion criteria or treatment strategy based on the results of interim analysis, 3) a multilevel endpoint system that integrates traditional clinical endpoints such as progression-free survival and lung function improvement rate with mechanistic endpoints such as the degree of p-Smad2/3 signal inhibition, changes in the immune cell composition, and imaging fibrosis score. Furthermore, liquid biopsy and molecular imaging technology can be used for dynamic monitoring and early prediction of treatment response and provide a real-time basis to evaluate efficacy (205). A “mechanism-clinical” dual-endpoint trial design can enhance the interpretability of results, accelerate the process of confirmatory research, and improve the overall efficiency of translational research.

### 7.6 Comprehensive considerations of risk management and ethical dimensions

Given the significance of TGF-β targeted therapy, it is essential to fully consider its core role in tissue homeostasis and immune balance, as well as the multiple potential risks associated with targeted intervention in terms of safety. Immune-related adverse reactions include autoimmune abnormal activation and exacerbation of chronic inflammatory states (206); tissue repair disorders manifest as delayed wound healing and impaired tissue regeneration capacity; what is more concerning is the long-term uncertainty, including the decline in immune surveillance function, which may lead to an increased risk of infection or secondary tumor occurrence (207). From an ethical perspective, special attention needs to be paid to precise individual risk–benefit assessment based on biomarkers, development of local delivery strategies and conditional activation prodrug technologies to improve treatment safety, and design of long-term follow-up mechanisms and real-world monitoring systems to comprehensively evaluate long-term effects. Establishing a systematic risk prediction model and active drug safety monitoring system is a necessary prerequisite for promoting the clinical translation of TGF-β targeted therapy and also helps to formulate reasonable treatment guidelines and ethical norms. Considering all these aspects, building a complete innovative chain of TGF-β research requires following a three-stage implementation route: the first stage (mechanism decoding), using multi-omics and spatial technologies to map microenvironment-specific signaling networks and identify key regulatory nodes and operable targets (208); the second stage (experimental verification), through gene editing, organoids, and humanized models to verify the target mechanism and comprehensively evaluate the pharmacological inhibitory effect and potential toxicity (209); and the third stage (clinical implementation), promoting stratified clinical trials based on biomarkers, developing combined strategies and precise delivery technologies, and ultimately achieving truly individualized treatment (210). Forming a complete closed loop of “mechanism-model-clinical” not only can establish a repeatable, verifiable, and transferable research paradigm, but will also significantly promote the substantive progress of TGF-β-targeted therapy from experimental research to clinical application, ultimately benefiting a large number of patients.

## 8 Conclusions

By systematically reviewing existing evidence, this article clearly states that the output of TGF-β’s functions does not follow a simple linear relationship; instead, it shows a high degree of context dependence. This characteristic depends on the combined action of various factors, including specific cell types, the dynamic balance of signaling pathways, the composition of the microenvironment factors, and different stages of disease development. At the molecular mechanism level, the classic Smad pathway and non-classical non-Smad pathways (including MAPK, PI3K/AKT, and Rho/ROCK, etc.) together form the core framework of the TGF-β signaling network, and these pathways achieve a dynamic balance of functions through complex cross-talk and precise feedback regulatory mechanisms. Furthermore, at the systems biology level, TGF-β regulates immune cell differentiation (such as the balance between regulatory Treg and Th17 cells), affects the activation state of stromal cells, and reshapes the composition of the extracellular matrix, profoundly shaping the characteristics of the tissue microenvironment. Thus, TGF-β signaling plays a critical role in shaping disease progression and clinical outcomes across diverse pathological contexts.

From a clinical translational perspective, TGF-β can serve as a potential biomarker for various diseases and is a highly promising therapeutic target. However, how to effectively inhibit pathological effects while preserving its physiological functions and achieving precise regulation of the therapeutic window remains a core challenge and main bottleneck currently faced.

Based on the above understanding, for future research and clinical translation, we propose the following three key development directions: 1) firstly, in-depth understanding of the mechanism requires the use of advanced methods such as single-cell multi-omics technology, spatial transcriptomics analysis, and real-time dynamic imaging to systematically analyze the dynamic changes of the TGF-β signaling network in different microenvironments and establish verifiable causal hypotheses and predictive mathematical models; 2) construction of a precise stratified treatment system that integrates molecular biomarkers, imaging features, and clinical staging information; establishment of a multidimensional and quantitative stratified strategy; precise identification of patient populations; and formulation of individualized intervention plans; and 3) development of local targeted delivery systems and time-controlled therapies, and combined drug regimens based on pathway cross-talk mechanisms to broaden the therapeutic window and improve the sustainability of therapeutic effects. Therefore, TGF-β should no longer be simply regarded as a single inhibitory target but should be regarded as a systemic regulatory hub in immune homeostasis and disease progression. In summary, future research and practice can only transform basic scientific discoveries into reproducible and scalable clinical treatment plans by establishing a comprehensive framework of “mechanism analysis-stratified precision-intervention dynamization” for precise treatment and individualized management of immune-related diseases.
